# Examining the association between serum galactose-deficient IgA1 and primary IgA nephropathy: a systematic review and meta-analysis

**DOI:** 10.1007/s40620-023-01874-8

**Published:** 2024-03-01

**Authors:** Pedro Alves Soares Vaz de Castro, Arthur Aguiar Amaral, Mariana Godinho Almeida, Haresh Selvaskandan, Jonathan Barratt, Ana Cristina Simões e Silva

**Affiliations:** 1 Interdisciplinary Laboratory of Medical Investigation, Unit of Pediatric Nephrology, Faculty of Medicine, Federal University of Minas Gerais (UFMG), Belo Horizonte, Brazil; 2 The Mayer IgA Nephropathy Laboratories, University of Leicester, Leicester, UK; 3 Department of Cardiovascular Sciences, University of Leicester, University Road, Leicester, LE1 7RH UK

**Keywords:** IgA nephropathy, Gd-IgA1, Glycosylation, Biomarker

## Abstract

**Background:**

IgA nephropathy (IgAN) is a common primary glomerular disease. The *O*-glycosylation status of IgA1 plays a crucial role in disease pathophysiology. The level of poorly-*O*-galactosylated IgA1, or galactose-deficient IgA1 (Gd-IgA1), has also been identified as a potential biomarker in IgAN. We sought to examine the value of serum Gd-IgA1 as a biomarker in IgAN, by investigating its association with clinical, laboratory, and histopathological features of IgAN.

**Methods:**

The review followed Preferred Reporting Items for Systematic Reviews and Meta-Analyses (PRISMA) recommendations and was registered in PROSPERO (CRD42021287423). The literature search was conducted in PubMed, Web of Science, Cochrane, and Scopus, and the selected articles were evaluated for eligibility based on predefined criteria. The methodological quality of the studies was assessed using the Newcastle–Ottawa Scale. Statistical analysis was performed to calculate effect sizes and assess heterogeneity among the studies.

**Results:**

This review analyzed 29 out of 1,986 studies, conducted between 2005 and 2022, with participants from multiple countries. Gd-IgA1 levels were not associated with age and gender, while associations with hypertension, hematuria, and proteinuria were inconsistent. In the meta-analyses, a correlation between serum Gd-IgA1 and estimated glomerular filtration rate was identified, however, the relationships between Gd-IgA1 levels and chronic kidney disease (CKD) stage and progression to kidney failure were inconsistent.

**Conclusions:**

Serum Gd-IgA1 levels were not associated with validated prognostic risk factors, but were negatively correlated with kidney function. Further research in larger studies using standardized assays are needed to establish the value of Gd-IgA1 as a prognostic risk factor in IgAN.

**Graphical abstract:**

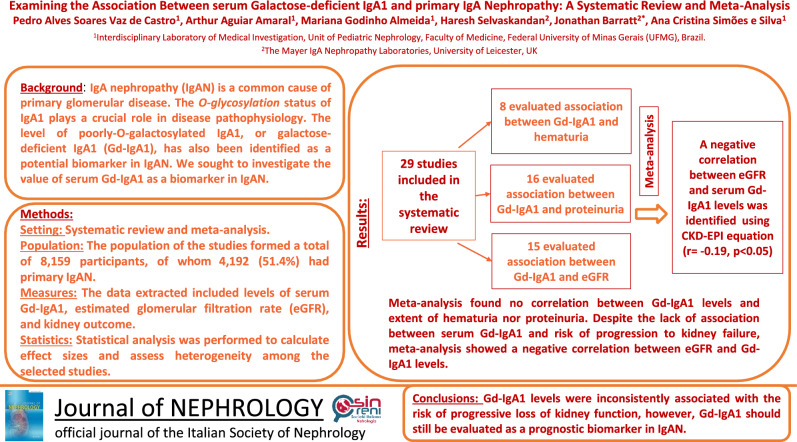

**Supplementary Information:**

The online version contains supplementary material available at 10.1007/s40620-023-01874-8.

## Introduction

IgA nephropathy (IgAN) is a frequent cause of primary glomerulonephritis and chronic kidney disease (CKD) worldwide [1]. Primary IgAN has a variable clinical course ranging from mild disease to kidney failure, with those of Asian ancestry displaying faster progression to kidney failure compared to those of European ancestry [2, 3].

Although the pathogenesis of IgAN is still under investigation, specific IgA1 *O*-glycoforms likely play a key role. Typically, the hinge region of IgA1 contains *O*-glycans comprising *N*-acetylgalactosamine (GalNAc) and galactose (Gal) and their sialylated forms. B cells in individuals with IgAN exhibit lower levels of core 1 β1,3-galactosyltransferase, an enzyme responsible for attaching galactose to GalNAc, as well as the molecular chaperone (Cosmc) necessary for stabilizing galactosyltransferase, as recently reviewed elsewhere [4]. Consequently, IgA1 in IgAN can have *O*-glycans with low levels of galactose at the hinge region, which consist of only GalNAc or sialylated GalNAc [5–7]. These *O*-glycans may act as auto-antigens, triggering the production of *O*-glycan-specific autoantibodies which can amplify IgA immune complex formation in the circulation and potentiate mesangial IgA deposition. Deposited immune complexes induce variable glomerular injury [8, 9] through mesangial cell activation, inflammatory cell recruitment and activation of the alternative and lectin pathways of complement [10–14].

Identifying those patients at greatest risk of progressive kidney failure is challenging. Well validated predictive factors for kidney failure include hypertension, proteinuria, kidney function, and kidney biopsy histomorphometry [15]. Histomorphometric lesions are evaluated using the MEST-C score, and include Mesangial hypercellularity, Endocapillary hypercellularity, Segmental glomerulosclerosis, interstitial fibrosis/Tubular atrophy and Crescents [16, 17]. These factors have been combined in the International IgA nephropathy risk prediction tool which allows calculation of an individual’s risk of a doubling of serum creatinine or kidney failure within 7 years of their kidney biopsy [18]. It is widely acknowledged that the precision of this tool could be improved and that there is a pressing need for new biomarkers in IgAN to not only improve prognostication but also treatment selection, and monitoring response to treatment [19].

Numerous biomarkers have been proposed, including mannose-binding lectin [20], soluble CD89-IgA complexes [12], and the IgA1/C3 ratio [21–23]. Many studies have reported higher levels of circulating Gd-IgA1 in IgAN compared to other kidney diseases and healthy subjects in several populations [24–29]. Gd-IgA1 levels have been reported to associate with histomorphometric lesions and kidney outcomes [30]. However, data are limited by small sample sizes and inconsistent findings [31–33]. The utility of Gd-IgA1 levels to predict prognosis is unclear with a number of conflicting studies [33, 34]. In this review and meta-analysis, our objective was to evaluate the evidence concerning Gd-IgA1 as a potential disease-specific prognostic biomarker for IgAN.

## Methods

### Protocol design and registration

This systematic review was registered in the International Prospective Register of Systematic Reviews (PROSPERO, CRD42021287423), and in the Open Science Frameworks (OSF, DOI: 10.17605/OSF.IO/6WXM5) and followed the Preferred Reporting Items for Systematic Reviews and Meta-Analyses (PRISMA) recommendations [35].

### Information sources and search strategies

Three authors independently performed a systematic search of the literature in PubMed, Web of Science, Cochrane, and Scopus by using the keywords “IgA Nephropathy”, “Berger’s Disease”, “Immunoglobulin A nephropathy” and similar entry terms collected from the Medical Subject Headings (MeSH) between the 5th and 27th of November, 2021. The search was updated on August 8th, 2023. The search strategy is shown in Table 1.

**Table 1:** Search strategies for database research

Database	Search Strategy
PubMed, Cochrane, Scopus	((("iga nephropathy") OR (Glomerulonephritis, IGA) OR (Glomerulonephritides, IGA) OR (Berger's Disease) OR (Bergers Disease) OR (IGA Glomerulonephritis) OR (IGA Nephropathy) OR (Immunoglobulin A Nephropathy) OR (Nephropathy, Immunoglobulin A) OR (Nephritis, IGA Type) OR (IGA Type Nephritis) OR (Nephropathy, IGA) OR (Berger Disease) OR (Iga Nephropathy 1) OR (Nephropathy 1, Iga)) AND ((Immunoglobulin A) OR (IgA) OR (IgA Antibody) OR (Antibody, IgA) OR (IgA1))) AND ((“Gd-IgA1”) OR (“poorly O galactosylated”) OR (“galactose deficient”) OR (“aberrantly glycosylated”) OR (“aberrantly galactosylated”) OR (Glycosylation) OR (Glycosylations Protein) OR (Glycosylation) OR (Glycosylation, Protein) OR (Glycosylations, Protein) OR (Protein Glycosylations))
Web of Science	((("iga nephropathy") OR (Berger's Disease) OR (Bergers Disease) OR (IGA Glomerulonephritis) OR (IGA Nephropathy) OR (Immunoglobulin A Nephropathy) OR (IGA Type Nephritis) OR (Berger Disease) OR (Iga Nephropathy 1)) AND ((Immunoglobulin A) OR (IgA) OR (IgA Antibody) OR (IgA1))) AND ((“Gd-IgA1”) OR (“poorly O galactosylated”) OR (“galactose deficient”) OR (“aberrantly glycosylated”) OR (“aberrantly galactosylated”) OR (Glycosylation) OR (Glycosylations Protein) OR (Glycosylation) OR (Glycosylation, Protein) OR (Glycosylations, Protein) OR (Protein Glycosylations))

### Eligibility criteria

Observational studies (case–control, cohort, and cross-sectional) and clinical trials were included. Included studies had to have patients with a kidney biopsy-confirmed diagnosis of primary IgAN in whom Gd-IgA1 levels had been measured. Articles published in English, Spanish, French, and Portuguese were eligible and no time restrictions were imposed. Exclusion criteria included studies investigating kidney diseases other than IgAN and recurrent IgAN post kidney transplant.

### Study selection and data extraction

After removing duplicates, two authors independently selected the articles by title and abstract, according to the predefined inclusion and exclusion criteria. In case of disagreement, a third author, an expert in the field, was consulted.

Full texts of the selected articles were gathered for complete evaluation. The following data were extracted: authorship, year, location, number of participants, objective and design, gender, age group, method of Gd-IgA1 measurement, and results obtained. Clinical and laboratory characteristics were also extracted and included: levels of serum Gd-IgA1, measures of creatinine, estimated glomerular filtration rate (eGFR), hematuria, 24-h proteinuria or protein/creatinine ratio in spot urine, blood pressure, and kidney outcome.

### Methodological quality evaluation

Two authors independently analyzed the methodological quality of the selected studies. Based on the Newcastle–Ottawa Scale (NOS) for cross-sectional studies, case–control studies, and cohort studies, three criteria were evaluated [36]: (i) selection (sample representativeness, sample size, non-respondents, and determination of exposure); (ii) comparability (control of confounding factors); and (iii) outcome (evaluation of results and statistical tests adopted). Studies with NOS scores 0–3, 4–6 and 7–9 (or 7–10 for cross-sectional studies) were considered as low, moderate and high quality, respectively.

### Statistical analysis

All analyses were performed using R software, version 4.1.0 (R Foundation for Statistical Computing, Vienna, Austria). Effect sizes were initially combined across studies using a fixed-effect model to obtain the summary estimate of effect size of the associations between serum Gd-IgA1 levels and pre-specified variables. Prior to combination, each effect size was transformed into a Fisher *z* score to normalize the distribution of *r* and make the variance independent of the population correlation. The weighted Fisher *z*-transformation, 95% confidence intervals (CI) and summary estimate were calculated. A weighted estimate effect size* r* was obtained on the assumption that all results were from the same population. Cochran’s Q test and *I*^*2*^ statistics were employed to quantify the heterogeneity among the results of the selected studies to test this assumption, with the significance level set at *p < *0.05. The degree of heterogeneity was interpreted according to the range of *I*^*2*^ as follows: 0–40%, likely not relevant; 30–60%, moderate; 50–90%, significant; and 75–100% substantial. Funnel and Baujat’s plots [37] were used to assess the heterogeneity of each study individually for all the meta-analyses (Supplementary Material 1).

## Results

**Fig. 1:**
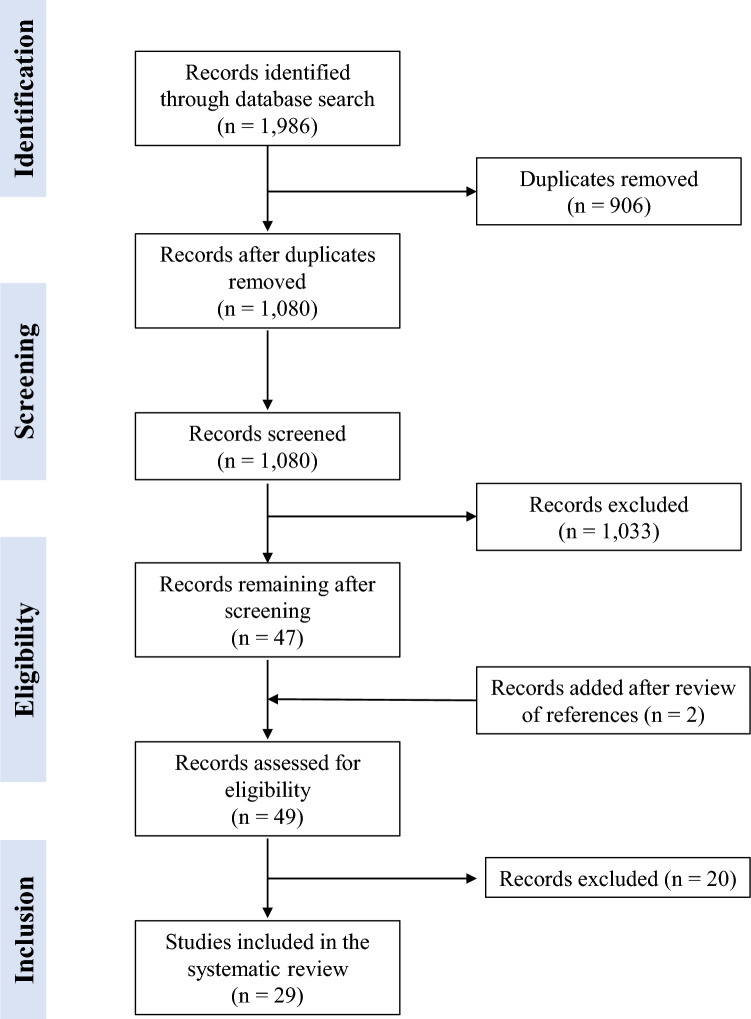
Flowchart of scientific article selection from the databases for the systematic review examining the ability of galactose-deficient IgA1 (Gd-IgA1) levels to predict prognosis in IgA nephropathy

The search strategy recovered 1,986 studies. After screening the title and abstract, 47 studies were selected for full-text reading and 1,938 were excluded (906 for duplication and 1,033 for not including the study question). Two more studies were included after review of references. Of 49 studies assessed, 29 met the eligibility criteria and were included in this systematic review [21, 24–27, 31, 33, 34, 38–58]. The publishing period ranged from 2005 [38] to 2022 [55, 56]. The detailed selection process is displayed in Fig. 1.

Regarding study design, four (13.7%) were case–control [25, 34, 38, 45], fourteen (48.2%) were cohort [21, 24, 31, 33, 39, 40, 46, 47, 49, 51, 53, 55, 56, 58], and eleven (37.9%) were cross-sectional [26, 27, 41–44, 48, 50, 52, 54, 57]. The studies were from ten different countries, including China (31.0%) [21, 26, 33, 38, 44, 48, 53, 57, 58], Japan (31.0%) [25, 41–43, 45, 47, 50, 54], United States of America (17.2%) [24, 27, 31, 40, 45], Italy (6.8%) [31, 45], Poland (6.8%) [46, 56], Spain (6.8%) [52, 55], South Korea (3.4%) [51], United Kingdom (3.4%) [58], India (3.4%) [34], and France (3.4%) [39]. Only three (10.3%) studies had patients from multiple countries [31, 45, 58]. The number of participants ranged from 50 [42] to 1418 [21], with a total of 8,159 participants, of whom 4,192 (51.4%) had primary IgAN. Thirteen (44.8%) studies included 990 kidney disease controls (mainly patients with Henoch-Schönlein nephritis, secondary IgAN, IgA vasculitis, minimal-change nephrotic syndrome and others) [25, 34, 43, 45–47, 50, 51, 53–57], ranging from 26 [46] to 205 [45] patients. Twenty-five studies had 2,943 healthy subjects [21, 24–27, 31, 33, 34, 38–41, 43–46, 48–51, 53, 54, 56–58], ranging from 20 [38, 41] to 638 [58] participants. One study also evaluated 34 relatives of patients with IgAN [27]. Unfortunately, none of the included studies compared the levels of Gd-IgA1 between patients with active and inactive IgAN.

### Methods for measuring Gd-IgA1 levels

All studies measured Gd-IgA1 levels using an enzyme-linked immunosorbent assay (ELISA), while one study also measured Gd-IgA1 levels with liquid chromatography-mass spectrometry [49]. The ELISA technique varied among the studies. Eight studies (27.5%) used the monoclonal antibody KM55 ELISA [34, 46–48, 51, 55–57], 17 studies (58.6%) used the *Helix aspersa-*based (HA) lectin ELISA [24–27, 31, 33, 39–43, 45, 49, 50, 52, 53, 58], 4 studies (13.7%) used the *Helix pomatia*-based (HPA) lectin ELISA [21, 52–54], and 2 studies (6.9%) used the *Vicia villosa*-based ELISA [38, 44].

### Gd-IgA1 may associate with ancestry but not with gender and age

Data on gender of controls were incomplete in 8/29 studies [21, 26, 27, 31, 45, 46, 57, 58]. Twenty one studies had gender information for all controls, in six there was a predominance of females [38, 42, 43, 47, 50, 52]. Gender data for patients with IgAN were incomplete in 3/29 studies [45, 46, 56]. Twenty six studies had gender information for all IgAN patients, with 2,421 (57.7%) being male. In 9/26 studies there was a predominance of females [25, 27, 38, 42, 43, 49, 50, 57]. No study demonstrated an association between gender and serum Gd-IgA1 levels in patients with IgAN or control populations.

**Fig. 2:**
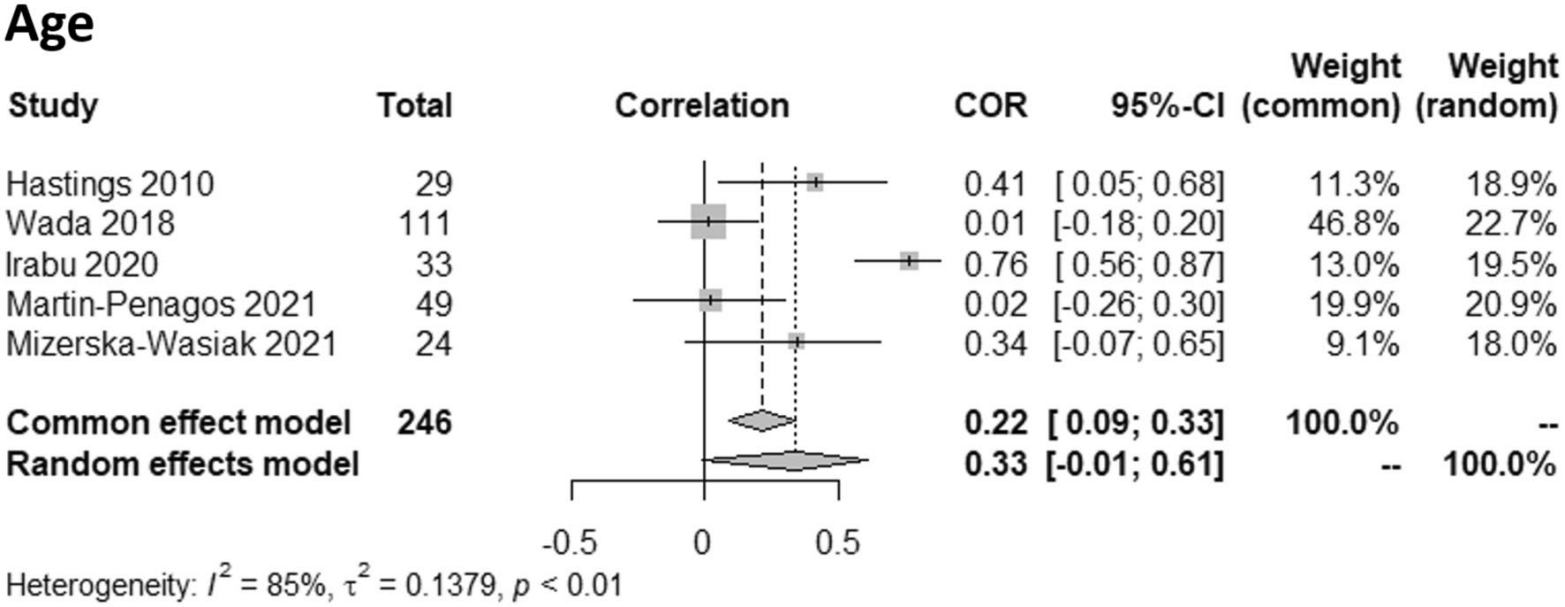
Meta-analysis of the correlation between age and levels of serum galactose-deficient IgA1 (Gd-IgA1) in patients with IgA nephropathy

Four of the 29 studies did not present complete information on the age of patients with IgAN [27, 31, 45, 58]. Eight studies included pediatric patients [27, 31, 40, 44, 46, 50, 53, 55]. A significant difference in the age of IgAN patients and controls was found in ten studies [34, 39, 41, 46, 47, 51, 53–56]. Eleven studies tested whether there was a correlation between age and serum Gd-IgA1 [27, 31, 33, 34, 39, 46, 47, 49, 50, 54, 55], with three detecting a positive correlation [40, 50, 56]. In the meta-analysis, no correlation between age and serum Gd-IgA1 levels was found using the random effects model, and studies showed a significant heterogeneity (*I*^*2*^ = 82%) (Fig. 2).

Only four studies evaluated whether serum Gd-IgA1 levels were associated with ancestry [27, 31, 40, 58]. In 3/4 of these studies serum Gd-IgA1 levels did not differ between patients with IgAN of different ancestries. However, in the only study to measure serum Gd-IgA1 levels in Chinese and White patients in the same laboratory with the same assay, White IgAN patients had significantly higher serum Gd-IgA1 levels [58]. In a separate study, healthy White subjects had higher serum Gd-IgA1 levels than healthy African American subjects [27].

### Gd-IgA1 does not associate with hypertension

Seven studies, including 3,003 participants, investigated the association between hypertension and serum levels of Gd-IgA [21, 26, 33, 39, 46, 48, 55]. Five of these studies (1,101 participants) reported no association between blood pressure and Gd-IgA1 levels [26, 33, 46, 48, 55]. Two studies did, however, report an indirect association between hypertension and Gd-IgA1 levels [21, 32]. In one study of 157 patients, for whom an absolute renal risk of dialysis or death was calculated using the number of risk factors present at diagnosis (which included hypertension), proteinuria higher than 1 g/day and kidney biopsy features, patients with a higher absolute renal risk of dialysis had both higher Gd-IgA1 levels and a higher prevalence of hypertension [32]. In the second study, patients with higher Gd-IgA1/C3 ratios at the time of kidney biopsy also had higher blood pressures [21].

### Gd-IgA1 is not correlated with hematuria

Only one of the eight studies [25, 42, 44, 46, 48, 55, 56] that tested an association between Gd-IgA1 and severity of hematuria reported a significant association [42]. These studies included 769 participants, of whom only 50 subjects were included in the study reporting an association [42]. The remaining seven studies (total of 719 patients) found no association between Gd-IgA1 and hematuria.

**Fig. 3:**
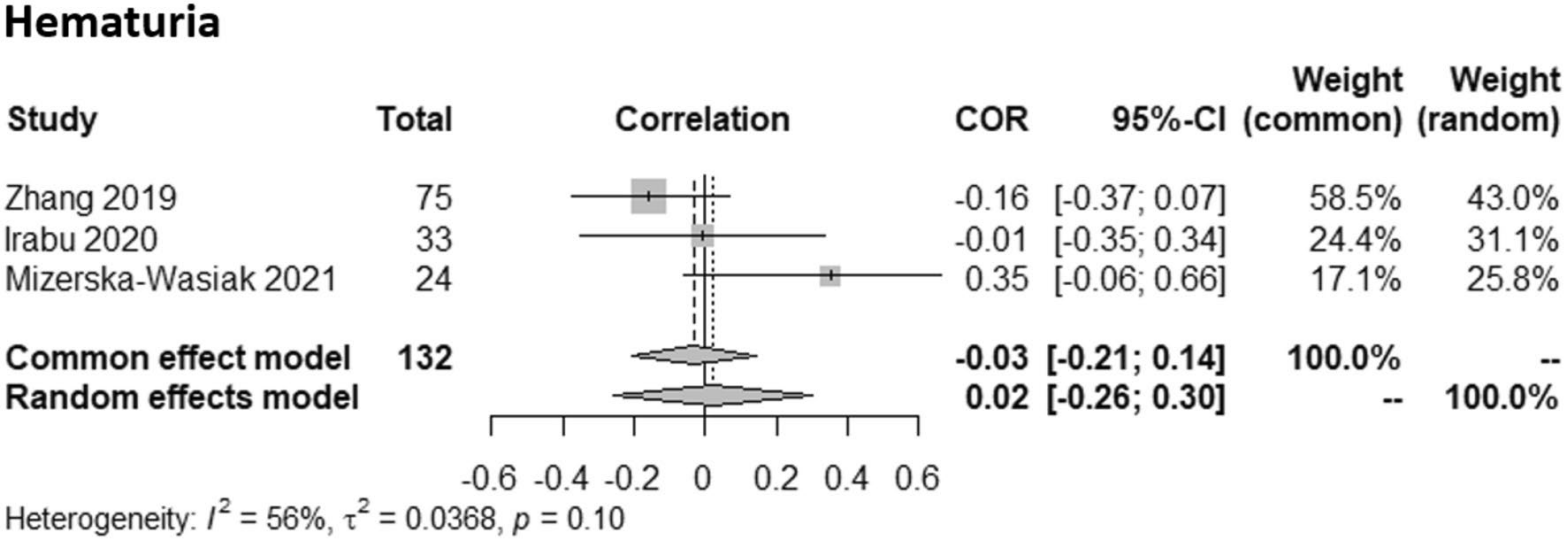
Meta-analysis of the correlation between levels of hematuria and serum galactose-deficient IgA1 (Gd-IgA1) in patients with IgA nephropathy

A correlation meta-analysis was conducted including all studies with quantitative hematuria data (number of erythrocytes per high power field) and serum Gd-IgA1 levels [48, 50, 56]. In both common effect and random effects models, no correlation was found (Fig. 3).

### Gd-IgA1 is not associated with proteinuria

Sixteen studies examined whether there was a correlation between proteinuria and Gd-IgA1 levels, and these studies included 3,552 patients [26, 31, 33, 34, 40, 42, 45–48, 50, 51, 53, 55–57]. Four studies, including 795 patients, found a positive correlation [42, 45, 50, 57]. Twelve studies, including 2,757 patients, did not find an association [26, 31, 33, 34, 40, 46–48, 51, 53, 55, 56].

**Fig. 4:**
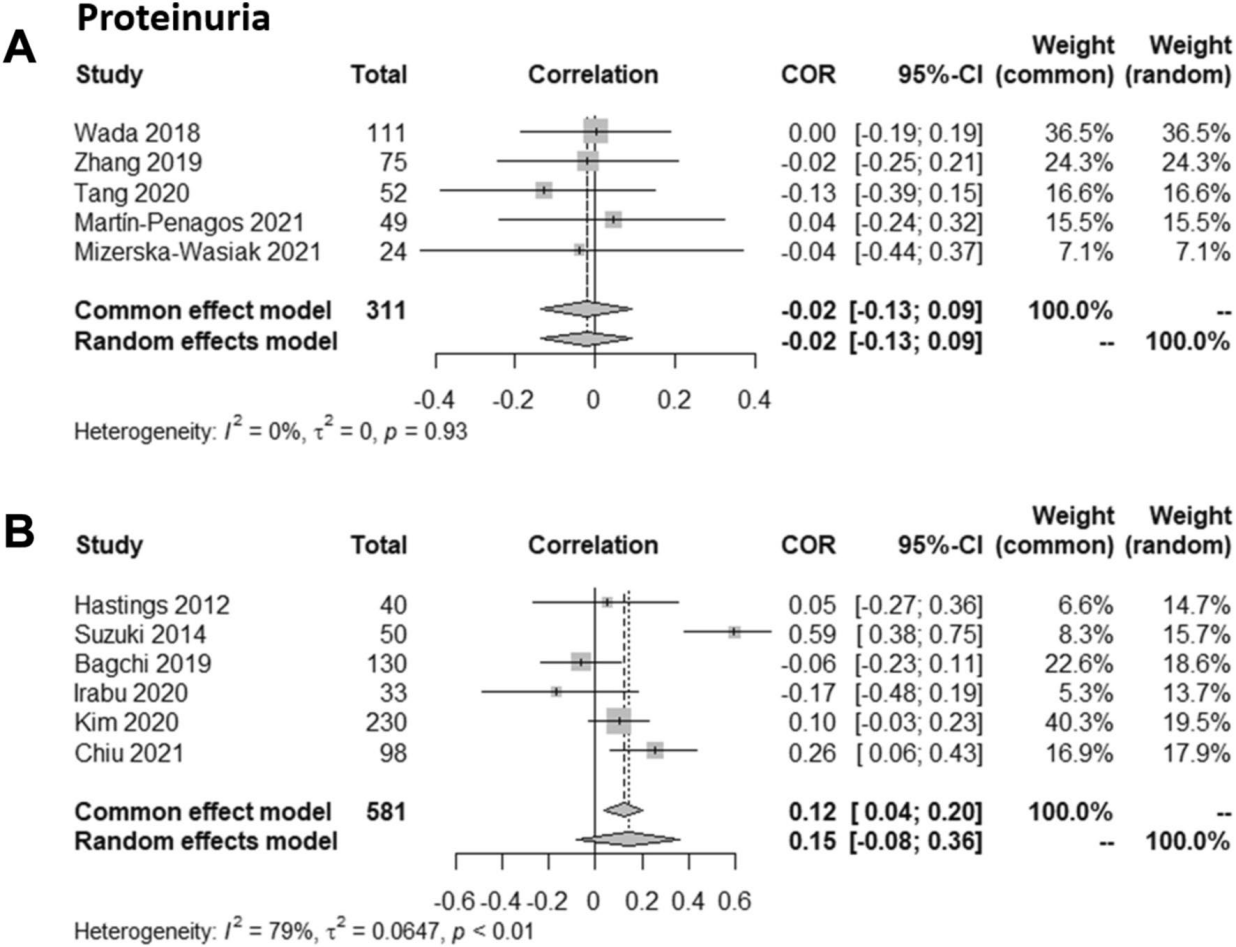
Meta-analyses of the correlation between proteinuria and serum galactose-deficient IgA1 (Gd-IgA1) in patients with IgA nephropathy. **A** Studies that evaluated 24 h proteinuria and **B** studies that evaluated urine protein creatinine ratio

Two separate correlation meta-analyses were performed based on the method used to measure proteinuria: 24-h proteinuria or urine protein-to-creatinine ratio (UPCR). Not all studies provided sufficient information for the analyses. In both meta-analyses no correlation between proteinuria and serum Gd-IgA1 levels was found (Fig. 4).

In the study by Berthoux et al*.* when absolute renal risk was calculated, patients with a higher absolute renal risk had higher Gd-IgA1 levels and higher levels of proteinuria [39].

### Gd-IgA1 is correlated with eGFR in patients with IgAN

Fifteen studies determined whether there was a correlation between eGFR and Gd-IgA1 levels, including a total of 3,571 patients [24, 26, 27, 31, 33, 34, 46–48, 51, 53, 55–57]. Different equations were used to determine eGFR. The Modification of Diet in Renal Disease (MDRD) equation was used in five studies [24, 31, 33, 34, 47], although two of these did not provide enough data to be included in the meta-analysis [31, 33]. Four studies in children used the Schwartz formula [34, 46, 53, 56]. Six studies used the Chronic Kidney Disease Epidemiology Collaboration (CKD-EPI) equation [48, 50–52, 55, 57]. One of these contained insufficient data and so was not included in the meta-analysis [52]. Seven studies, including 1,013 patients, reported a negative correlation between Gd-IgA1 levels and eGFR [46, 47, 50, 51, 55–57]. The remaining eight studies, including 2,558 patients, did not find a correlation.

**Fig. 5:**
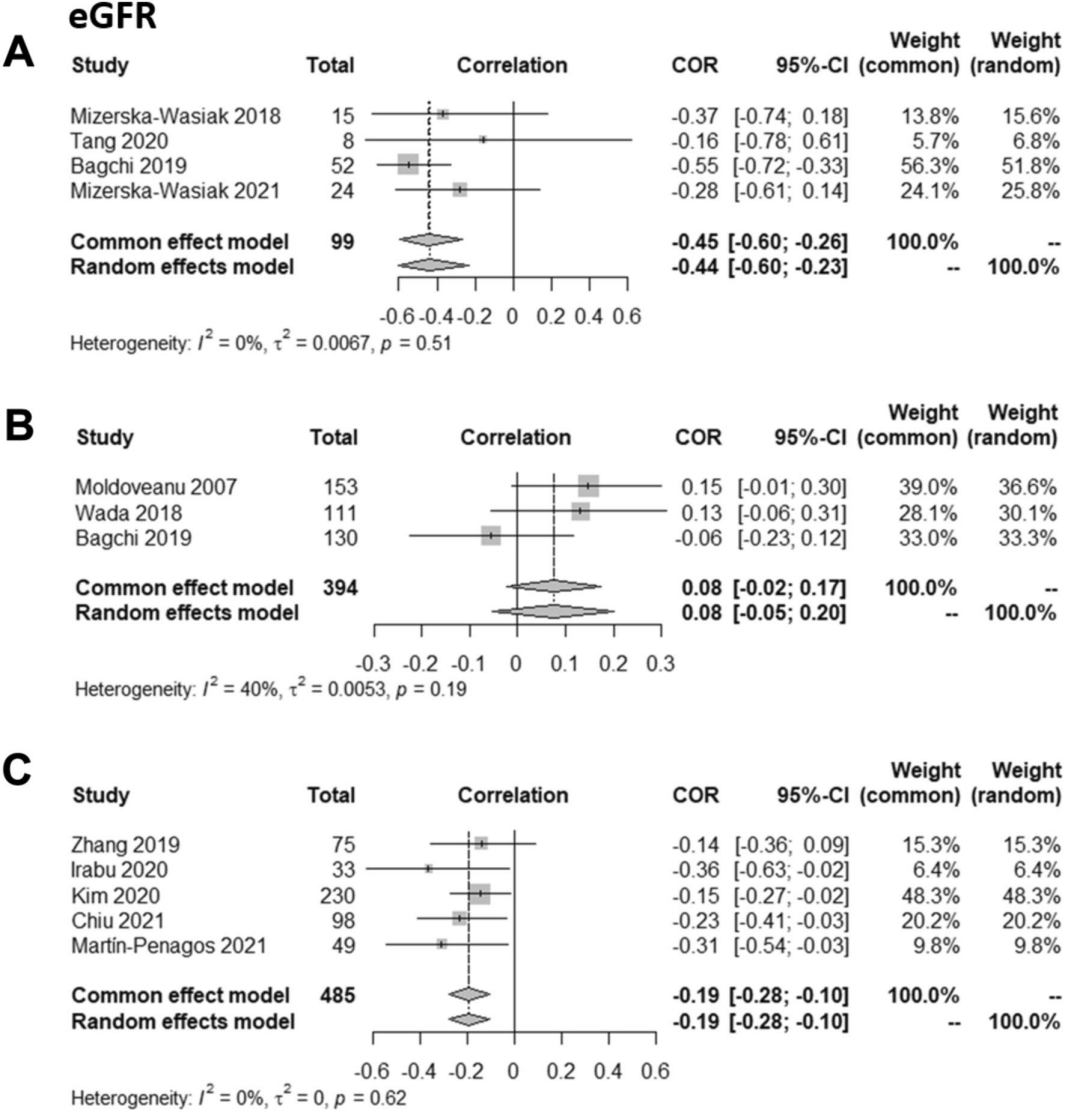
Meta-analyses of the correlation between estimated glomerular filtration rate (eGFR) and serum galactose-deficient IgA1 (Gd-IgA1) in patients with IgA nephropathy using different eGFR formulas. **A** Studies that evaluated eGFR calculated using the Schwartz formula, **B** studies that evaluated eGFR calculated using the Modification of Diet in Renal Disease (MDRD) equation and **C** studies that evaluated eGFR calculated using the Chronic Kidney Disease Epidemiology Collaboration (CKD-EPI) 2012 equation

In children, the meta-analysis showed a negative correlation between eGFR (Schwartz formula) and levels of Gd-IgA1 (Fig. 5A). By contrast, in adult patients the meta-analysis results were discordant. The meta-analysis of studies using the MDRD eGFR did not show a correlation (Fig. 5B), and displayed high heterogeneity (*I*^*2*^ = 40%), while a negative correlation between eGFR and serum Gd-IgA1 levels was found when the CKD-EPI equation was reported using both common and random effects models (Fig. 5C).

Two other studies, not included in the analyses, indirectly assessed this relationship [49, 52]. Dotz et al. found that patients with different patterns of IgA *O*- and *N-*glycosylation measured with liquid chromatography-mass spectrometry had different baseline eGFRs [49]. Medrano et al. measured the levels of Gd-IgA1 using 3 different lectin-binding ELISAs in serum samples treated and not-treated with neuraminidase [52]. In this study there was a stronger association between Gd-IgA1 levels and eGFR when the serum samples were not treated with neuraminidase.

### Gd-IgA1 is not associated with CKD stage progression

Six studies, including 1187 participants, examined the association between Gd-IgA1 levels and risk of CKD progression (defined by sustained > 30% irreversible decline in eGFR [34] or progression of CKD stage according to the KDIGO guideline [31, 33, 39, 51, 55]). Two studies, including 395 patients, found that higher Gd-IgA1 levels were associated with a greater likelihood of CKD stage progression [51, 55]. By contrast, four studies including 792 patients did not find an association [31, 33, 34, 39].

### Gd-IgA1 and progression to kidney failure

Only six studies determined whether there was an association between Gd-IgA1 levels and progression to kidney failure [21, 33, 34, 40, 47, 58]. Hastings et al*.* found that two of three children who developed kidney failure had Gd-IgA1 levels above the 95th percentile for healthy children [40]. Zhao et al*.* [33] reported that the kidney survival rate was associated with serum Gd-IgA1 levels when serum Gd-IgA1 levels were placed into quartiles. Kidney survival at 1 and 3 years in the 1st (lowest), 2nd, 3rd and 4th (highest) Gd-IgA1 quartile levels were respectively: 100.0% and 96.9%, 100.0% and 91.8%, 100.0% and 92.2%, 98.6% and 88.6%. Wada et al. reported that serum Gd-IgA1 levels were significantly increased in patients at high or very high risk of kidney failure when compared to those at low risk [47]. Bagchi et al.[34] evaluated the probability of kidney survival at 12 and 48 months for patients with higher and lower serum Gd-IgA1 levels. While not statistically significant, reported kidney survival rates were 67.0% versus 80.1% and 39.6% versus 62.9%, respectively. Chen et al*.* reported that patients with higher Gd-IgA1 levels had a higher rate of eGFR decline (*p < *0.001) and that patients with higher quartile levels of Gd-IgA1/C3 ratio also had an increased risk of kidney failure [21]. Consistent with these observations, Gale et al. found that Gd-IgA1 levels were significantly higher (*p = *0.001) in progressors (defined as doubling of serum creatinine or needing renal replacement therapy) compared to nonprogressors (defined as serum creatinine < 1.35 mg/dl and < 20% increase over at least 5 years of follow-up) [58].

### Gd-IgA1 may be associated with histomorphometric features of the Oxford classification

Nine studies evaluated the association between Gd-IgA1 levels and the Oxford classification [21, 25, 34, 47, 48, 51, 53, 55, 56]. Four of these found an association (1,600 patients) [21, 47, 51, 55], with Wada et al.[47] and Kim et al.[51] reporting associations between increasing serum Gd-IgA1 levels and increasing tubular atrophy/interstitial fibrosis. Martín-Penagos et al. found that Gd-IgA1 levels were higher in patients with more mesangial hypercellularity and more extensive tubulointerstital inflammation and fibrosis atrophy (T2 > T1 > T0) [55]. Separately, in a multivariate analysis, Chen et al*.* found an association between the presence of endocapillary hypercellularity (E1), segmental glomerulosclerosis (S1), and tubular atrophy/interstitial fibrosis (T1/2) and Gd-IgA1/C3 ratio [21].

### Quality assessment

With respect to the quality assessment, studies were divided according to their design and appropriate NOS [36] (Table 2). The overall quality score of the cohort studies was 7.25/9, which was considered moderate quality. Most cohort studies lost points based on the comparability criterion. Concerning cross-sectional studies, the overall quality score was 5.58/10, which was again considered moderate quality. Most cross-sectional studies similarly lost points based on the comparability criterion. Case–control studies had an overall quality score of 3.5/9, which was considered low quality. Most of the included case–control studies lost points based on the selection and outcome criteria.

**Table 2:** Quality assessment according to the Newcastle–Ottawa Scale[36]

Studies	Criteria			Total (0–9 points)
	Selection (0–4 points)	Comparability (0–2 points)	Outcome (0–3 points)	
Cohort studies				
Camilla et al. 2011	4	2	3	9
Berhoux et al. 2012	4	1	3	8
Hastings et al. 2012	3	1	2	6
Gale et al. 2017	4	2	3	9
Wada et al. 2018	4	1	2	7
Chen et al. 2019	2	2	2	6
Kim et al. 2020	3	1	2	6
Mizerska-Wasiak et al. 2021	3	1	3	7

Cross-sectional studies	Selection (0–5 points)	Comparability (0–2 points)	Outcome (0–3 points)	Total (0–10 points)
Moldoveanu et al. 2007	2	0	3	5
Lin et al. 2009	2	0	3	5
Hastings et al. 2010	3	0	3	6
Satake et al. 2014	2	0	3	5
Suzuki et al. 2014	2	0	3	5
Yanagawa et al. 2014	3	2	3	8
Jiang et al. 2015	2	0	3	5
Zhang et al. 2019	2	0	3	5
Dotz et al. 2020	3	0	3	6
Wang et al. 2020	2	2	3	7
Chiu et al. 2021	2	1	3	6
Martín-Penagos et al. 2021	2	2	3	7
Irabu et al. 2020	3	2	3	5
Medrano et al. 2020	2	0	3	5
Zhao et al. 2012	2	0	3	5
Mizerska-Wasiak, 2018	1	1	2	4
Tang et al. 2020	2	1	3	6

Case–control studies	Selection (0–4 points)	Comparability (0–2 points)	Outcome (0–3 points)	Total (0–9 points)
Shimozato et al. 2008	1	2	1	4
Suzuki et al. 2016	1	2	1	4
Bagchi et al. 2019	1	2	1	4
Xu et al. 2005	1	0	1	2

## Discussion

The aim of this study was to determine whether Gd-IgA1 levels could be used to evaluate disease activity and prognosis in patients with IgAN. In the majority of studies, patients with IgAN had higher Gd-IgA1 levels than healthy subjects and/or kidney disease controls. The analyzed studies did not find a significant difference in serum Gd-IgA1 levels between male and female patients with IgAN. While three studies reported a positive correlation between age and Gd-IgA1 levels [40, 50, 56], the meta-analysis found no correlation. The relationship between Gd-IgA1 levels and ancestry is incompletely understood, however, quantitative trait genome-wide association studies have identified that *O*-galactosylation of IgA1 is associated with a common variation in *C1GALT1* which encodes the galactosyltransferase enzyme that catalyses addition of galactose to GalNAc at the IgA1 hinge region. Consistent with the frequency of the associated single nucleotide polymorphisms in individuals of European and Chinese ancestry, White IgAN patients have higher Gd-IgA1 levels than Chinese IgAN patients [58].

Microscopic hematuria, frequently detected during routine health screening, is a common first sign of IgAN [59, 60]. Despite hematuria being commonly thought of as a biomarker of glomerular inflammation, the extent of hematuria is not associated with Gd-IgA1 levels. The majority of the identified studies did not show a correlation between extent of hematuria and levels of Gd-IgA1 [25, 42, 44, 46, 48, 50, 55, 56], a finding that was confirmed in the meta-analysis. However, not all studies used the same cut-off values for hematuria and those studies that only evaluated hematuria with dipstick testing were not included in the meta-analysis.

Proteinuria is considered an early marker of glomerular damage, and an important prognostic biomarker in IgAN [61]. The extent of proteinuria is associated with disease progression and histological findings that indicate worse clinical outcomes [62, 63]. In the identified studies proteinuria was assessed differently, with some reporting 24 h proteinuria [47, 48, 53, 55, 56] and others UPCR [34, 40, 41, 50, 51, 57]. Furthermore, some studies did not report sufficient data to allow inclusion in the meta-analysis. The meta-analysis found no correlation between proteinuria and serum Gd-IgA1 levels. Proteinuria can be secondary to active glomerular lesions, triggered by Gd-IgA1-containing immune complex deposition, or chronic lesions such as glomerulosclerosis [45, 64]. Gd-IgA1 levels may, therefore, only associate with the extent of proteinuria early in the natural history of the disease before there is accumulation of significant kidney scarring.

The Oxford-MEST-C classification of IgAN includes five distinct histomorphometric features that independently predict the risk of kidney failure [65]. Only nine studies [21, 34, 47, 48, 51, 53, 55, 56] investigated the association of Gd-IgA1 levels with histological changes using this classification. Of these, four found an association between Gd-IgA1 levels and histomorphometric features, predominantly the T lesion [21, 46, 51, 55].

Eight studies evaluated serum Gd-IgA1 levels in children [27, 31, 40, 44, 46, 50, 53, 56]. Like adults, children with IgAN had significantly higher levels of Gd-IgA1 than non-IgAN glomerular disease controls [50] and healthy subjects [44]. While proteinuria is more often a marker of glomerular proliferative lesions in children with IgAN [66], there was no consistent relationship between serum Gd-IgA1 levels and extent of proteinuria in children. In addition, two studies reported that Gd-IgA1 levels were not associated with histological lesions or clinical outcomes in children with IgAN [40, 46]. While the extent of glomerular changes is markedly different between children and adults, [65, 67, 68], we identified no studies that compared Gd-IgA1 levels and histological changes in both children and adults.

Seven studies evaluated the association between serum Gd-IgA1 levels and risk of CKD progression [26, 31, 33, 34, 44, 51, 55], with six studies reporting the association between serum Gd-IgA1 levels and risk of kidney failure [21, 33, 34, 40, 47, 58]. The studies that reported CKD progression failed to identify a significant association, and due to the lack of sufficient data a meta-analysis was not possible. Those studies reporting CKD stage progression are likely to have low sensitivity for identifying an association between serum Gd-IgA1 levels and risk of progression due to the broad range of eGFRs included in each CKD stage [69]. Despite this lack of association a negative correlation was seen between levels of Gd-IgA1 and eGFR in children [70] and in adults (using the CKD-EPI equation) [71]. Gd-IgA1 levels were associated with progression to kidney failure in some of the reviewed studies [33, 47]. Gale et al. defined IgAN patients as “progressors” and “nonprogressors” and found that Gd-IgA1 levels were significantly higher, and the *C1GALT1* risk haplotype more frequent, in IgAN progressors.

While there is a general consensus that Gd-IgA1 has a pivotal role in the pathogenesis of IgAN [72], its role as a biomarker for risk of progression is complicated by the impact of confounding treatments and a potential varied role in determining loss of kidney function at different times in the natural history of the disease. It has also been reported that many first-degree relatives of patients with IgAN have comparably high levels of Gd-IgA1 for years without exhibiting kidney disease thereby implying that factors other than Gd-IgA1 determine the likelihood of developing IgAN [73]. Furthermore, mesangial deposition of Gd-IgA1 does not always lead to the development of clinical disease [74], and the inflammatory response to IgA deposition is highly heterogeneous [75]. In that sense, the presence of Gd-IgA1 would appear to not be the only cause of IgAN, but rather an important feature of the disease that plays a role in its pathophysiology.

A significant limitation of this analysis is that we had to compare and combine studies employing different methods of measuring serum levels of Gd-IgA1. Lectin-based assays were commonly used to measure Gd-IgA1. These lectins are purified from snails or plants and it is widely accepted that the *O*-glycan sensitivity of individual lectins can vary significantly between batches [76]. To reduce variability and increase reproducibility, a Gd-IgA1-specific monoclonal antibody (KM55) was developed [76] and has been used in several studies that measured Gd-IgA1 levels [46–48, 51, 55]. Although Gd-IgA1 levels appear similar regardless of the method used, there are few studies directly comparing these methods [76].

On reviewing the quality of the included studies, most had questionable quality according to NOS, especially in terms of comparability between groups. Case–control studies were of the poorest quality, with increased risks of bias in the selection process and analysis of outcomes. These biases likely significantly contributed to the variability of the results. For example, in the correlation meta-analysis of Gd-IgA1 levels and UPCR, one study alone contributed almost 15% of the heterogeneity and influence on the results [42].

On the basis of published literature the value of Gd-IgA1 as a prognostic biomarker in IgAN is uncertain. The vast majority of these studies have evaluated Gd-IgA1 as a biomarker without considering Gd-IgA1 levels in the context of existing validated biomarkers such as proteinuria, blood pressure, eGFR and MEST-C. To be clinically relevant it needs to be established whether Gd-IgA1 levels add prognostic precision beyond that achieved with the current KDIGO recommended approach using the International IgA nephropathy risk prediction tool. What is needed is a study that incorporates Gd-IgA1 levels into the prediction tool and evaluates whether prognostication has been improved, as has been undertaken with other biomarkers in IgAN [77, 78].

Several studies have investigated the role of Gd-IgA1 as a potential biomarker for IgAN progression. In this systematic review and meta-analysis, serum Gd-IgA1 levels were inconsistently associated with the risk of progressive loss of kidney function, however, there are sufficient data to justify continued evaluation of Gd-IgA1 as a prognostic biomarker in IgAN. More recently, Gd-IgA1 levels have been used to monitor the response to novel B-cell directed therapies, and data are emerging reporting significant reductions in Gd-IgA1 in association with decrease in proteinuria and stabilization of eGFR with some of these novel therapies [79], supporting continued study of Gd-IgA1 in IgAN.

## Supplementary Information

Below is the link to the electronic supplementary material.

Supplementary file1 (PDF 703 KB)
