# Supplementary material for: Examining the association between serum galactose-deficient IgA1 and primary IgA nephropathy: a systematic review and meta-analysis
Source: J Nephrol. 2024 Mar 1;37(8):2099–112. doi: 10.1007/s40620-023-01874-8 (PMC11649794; doi:10.1007/s40620-023-01874-8)
Supplement: Supplementary file 1 — Supplementary file1 (PDF 703 KB) [file 40620_2023_1874_MOESM1_ESM.pdf]

Examining the Association Between serum Galactose-deficient IgA1 and primary IgA Nephropathy: A Systematic Review and Meta-Analysis

Pedro Alves Soares Vaz de Castro[1], Arthur Aguiar Amaral[1], Mariana Godinho Almeida[1], Haresh Selvaskandan[2], Jonathan Barratt[2*], Ana Cristina Simões e Silva[1]

[1]Interdisciplinary Laboratory of Medical Investigation, Unit of Pediatric Nephrology, Faculty of Medicine, Federal University of Minas Gerais (UFMG), Brazil

[2]The Mayer IgA Nephropathy Laboratories, University of Leicester, UK

Conflict of Interest: None declared

Financial support. N/A

CORRESPONDENCE: Dr. Jonathan Barratt, Department of Cardiovascular Sciences, University of Leicester, University Road, Leicester, LE1 7RH, United Kingdom. Email: jb81@leicester.ac.uk

Supplementary material

Age

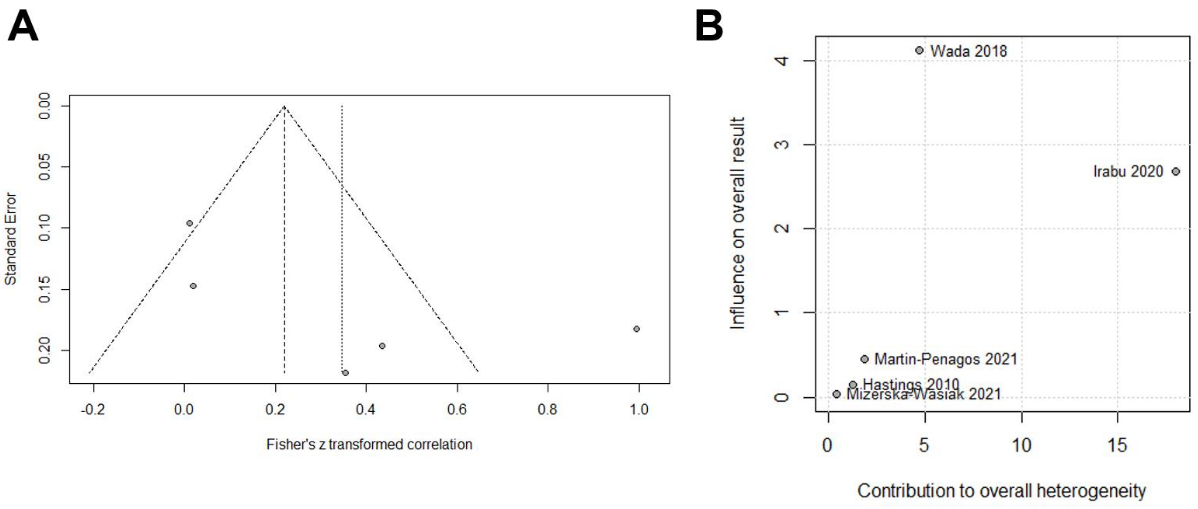

Figure S1. (A) Funnel plot and (B) Baujat's plot for studies included in the meta-analysis of correlation between participant's age and serum galactose deficient Gd-IgA1 (Gd-IgA1).

24h proteinuria

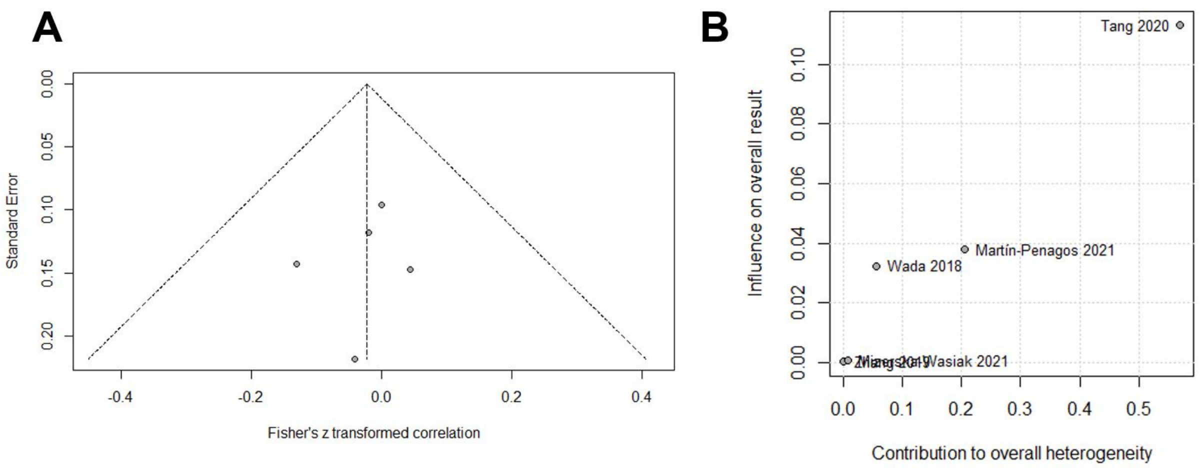

Figure S2. (A) Funnel plot and (B) Baujat's plot for studies included in the meta-analysis of correlation between 24h proteinuria and serum galactose deficient Gd-IgA1 (Gd-IgA1).

UPCR

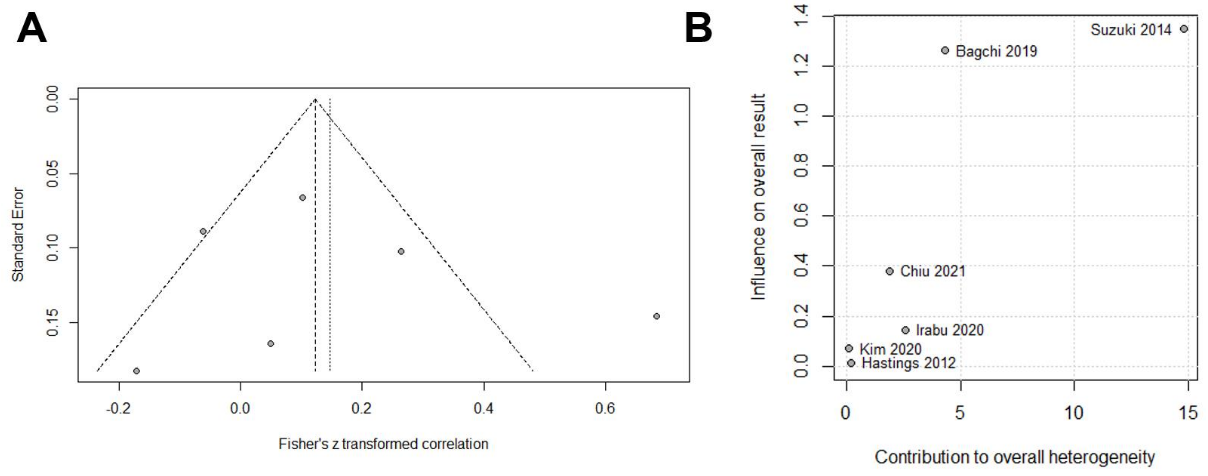

Figure S3. (A) Funnel plot and (B) Baujat's plot for studies included in the meta-analysis of correlation between urinary protein to creatinine ratio (UPCR) and serum galactose deficient Gd-IgA1 (Gd-IgA1).

CKD-EPI

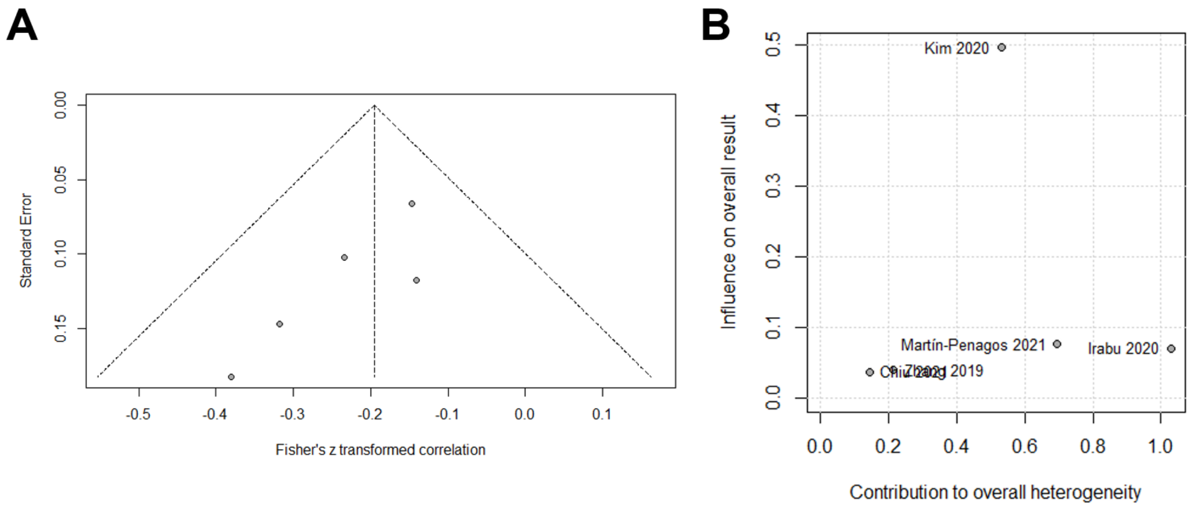

Figure S4. (A) Funnel plot and (B) Baujat's plot for studies included in the meta-analysis of correlation between estimated glomerular filtration rate (eGFR) using the CKD-EPI equation and serum galactose deficient Gd-IgA1 (Gd-IgA1).

MDRD

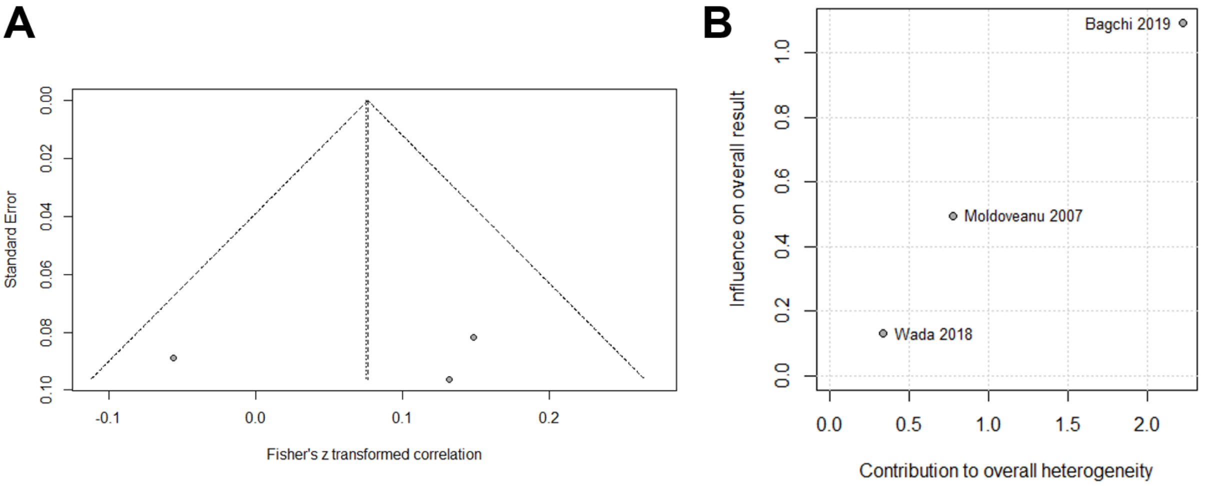

Figure S5. (A) Funnel plot and (B) Baujat's plot for studies included in the meta-analysis of correlation between estimated glomerular filtration rate (eGFR) using the Modification of Diet in Renal Disease (MDRD) equation and serum galactose deficient Gd-IgA1 (Gd-IgA1).

Schwartz

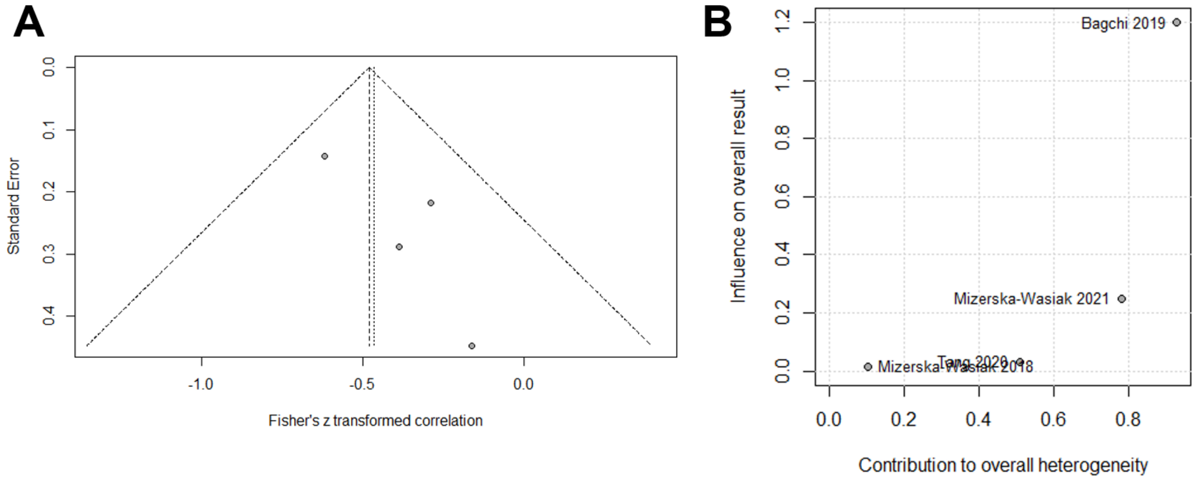

Figure S6. (A) Funnel plot and (B) Baujat's plot for studies included in the meta-analysis of correlation between estimated glomerular filtration rate (eGFR) using the Schwartz's equation and serum galactose deficient Gd-IgA1 (Gd-IgA1).

Hematuria

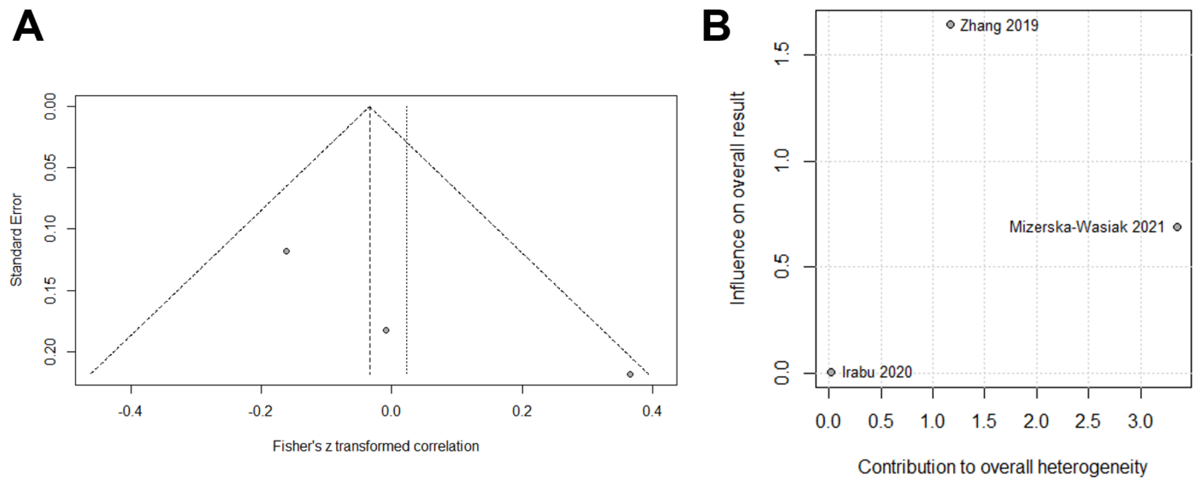

Figure S7. (A) Funnel plot and (B) Baujat's plot for studies included in the meta-analysis of correlation between hematuria and serum galactose deficient Gd-IgA1 (Gd-IgA1).
